# HCV Proteins Modulate the Host Cell miRNA Expression Contributing to Hepatitis C Pathogenesis and Hepatocellular Carcinoma Development

**DOI:** 10.3390/cancers13102485

**Published:** 2021-05-19

**Authors:** Devis Pascut, Minh Hoang, Nhu N. Q. Nguyen, Muhammad Yogi Pratama, Claudio Tiribelli

**Affiliations:** 1 Liver Research Center, Fondazione Italiana Fegato—ONLUS, Basovizza, 34149 Trieste, Italy; yogi.pratama@fegato.it (M.Y.P.); ctliver@fegato.it (C.T.); 2 Department of Microbiology and Immunology, Pasteur Institute of Ho Chi Minh City, Ho Chi Minh City 72408, Vietnam; hoangminh_bio@yahoo.com; 3 Center for Molecular Biomedicine, University of Medicine and Pharmacy at Ho Chi Minh, Ho Chi Minh City 72408, Vietnam; nnqnhu@ump.edu.vn

**Keywords:** HCV, hepatitis C, viral proteins, HCC, microRNA, miRNAs

## Abstract

**Simple Summary:**

According to the last estimate by the World Health Organization (WHO), more than 71 million individuals have chronic hepatitis C worldwide. The persistence of HCV infection leads to chronic hepatitis, which can evolve into liver cirrhosis and ultimately into hepatocellular carcinoma (HCC). Although the pathogenic mechanisms are not fully understood, it is well established that an interplay between host cell factors, including microRNAs (miRNA), and viral components exist in all the phases of the viral infection and replication. Those interactions establish a complex equilibrium between host cells and HCV and participate in multiple mechanisms characterizing hepatitis C pathogenesis. The present review aims to describe the role of HCV structural and non-structural proteins in the modulation of cellular miRNA during HCV infection and pathogenesis.

**Abstract:**

Hepatitis C virus (HCV) genome encodes for one long polyprotein that is processed by cellular and viral proteases to generate 10 polypeptides. The viral structural proteins include the core protein, and the envelope glycoproteins E1 and E2, present at the surface of HCV particles. Non-structural (NS) proteins consist of NS1, NS2, NS3, NS4A, NS4B, NS5a, and NS5b and have a variable function in HCV RNA replication and particle assembly. Recent findings evidenced the capacity of HCV virus to modulate host cell factors to create a favorable environment for replication. Indeed, increasing evidence has indicated that the presence of HCV is significantly associated with aberrant miRNA expression in host cells, and HCV structural and non-structural proteins may be responsible for these alterations. In this review, we summarize the recent findings on the role of HCV structural and non-structural proteins in the modulation of host cell miRNAs, with a focus on the molecular mechanisms responsible for the cell re-programming involved in viral replication, immune system escape, as well as the oncogenic process. In this regard, structural and non-structural proteins have been shown to modulate the expression of several onco-miRNAs or tumor suppressor miRNAs.

## 1. Introduction

Hepatitis C virus (HCV) is an enveloped, 9.6 kb single-stranded RNA, virus belonging to the Hepacivirus genus, with six major genotypes [1]. According to the last estimate by the World Health Organization (WHO), more than 71 million individuals have chronic hepatitis C worldwide. Horizontal blood-borne transmissions such as intravenous drug use, needle pricks, blood transfusion, and high-risk sexual practices, as well as vertical transmission from mother to infants are the main causes of HCV infection [2,3]. The persistence of HCV infection leads to chronic hepatitis, which can evolve into liver cirrhosis and ultimately into hepatocellular carcinoma (HCC). The interferon (IFN)/ribavirin-based antiviral therapy represented the eligible treatment against HCV for years despite being poorly tolerated and the sustained viral clearance was achieved in only a subset of HCV infections [4,5]. With the introduction of direct-acting antiviral (DAA) treatments for all HCV genotypes, sustained virological response (SVR) was observed in >90% of treated individuals with a consequent reduced risk of future complications [6]. However, achieving SVR does not completely eradicate the risk of developing HCC, as the long-term possibility remains for up to 8 years in cirrhotic patients with HCV who achieve SVR [7,8]. Several studies since 2016 have reported a significant concern about an unexpected high rate of HCC occurrence after DAA therapy, which led to a large debate whether or not patients with HCV have an increased risk to develop HCC after DAA treatment [9,10]. Considering that only one-per-third of patients with chronic HCV have received DAA treatments worldwide [11], the burden to prevent HCC development in this high-risk group remains a priority and understanding complex molecular networks that participate in HCC development in HCV patients can be a solid foundation to develop better prevention, surveillance, and treatment strategies.

Although the pathogenic mechanisms are not fully understood, it is well established that an interplay between host cell factors [12], including microRNAs (miRNA) [13], and viral components exist in all the phases of the viral infection and viral replication. Those interactions establish a complex equilibrium between host cells and HCV and participate in multiple mechanisms characterizing hepatitis C pathogenesis. Besides, HCV may promote the development of HCC through several mechanisms, which include: (1) persistent liver inflammation and immune-mediated oxidative stress damage from a chronic viral infection; (2) metabolic reprogramming leading to steatosis that further progresses into fibrosis and HCC; (3) intracellular oxidative stress damage induced by viral proteins; and (4) deregulation of cell signaling pathways by viral protein (HCV core, NS3, and NS5 A/B) [14].

HCV structural and non-structural proteins have drawn the main attention since their interactions with host cell factors in several pathways [12], such as immune escape, lipid metabolism, cell cycle regulation, cell proliferation, transcriptional regulation, and some of them are tightly associated with HCC development [15,16]. More recently, miRNAs have emerged as new intriguing players in the interaction between HCV and host cells.

Considering that these regulatory networks remain to be fully elucidated, the present review aims to describe the role of HCV structural and non-structural proteins in the modulation of cellular miRNA during HCV infection and pathogenesis, ultimately leading to HCC development (see Appendix A for further details about searching methodology).

## 2. HCV Proteins and Their Role in Infection

**Figure 1:**
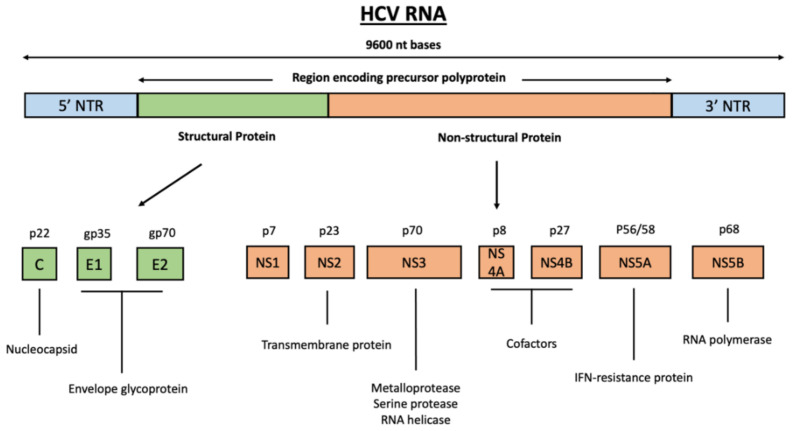
Proteins encoded by the HCV genome. HCV viral genome encodes one long polyprotein that is co- and post-translationally processed into mature structural and non-structural proteins. The viral structural proteins (green) include the core protein and envelope glycoproteins E1 and E2; the viral non-structural proteins (orange) consist of NS1, NS2, NS3, NS4A, NS4B, NS5A, and NS5B proteins.

HCV viral genome encodes for one long polyprotein that is co- and post-translationally processed by cellular and virally encoded proteases into mature structural and non-structural (NS) proteins (Figure 1) [17]. The viral structural proteins include the core protein, which is contained in the virus particle, and the envelope glycoproteins E1 and E2 on the surface of HCV particles [18]. The non-structural (NS) proteins consist of NS1, NS2, NS3, NS4A, NS4B, NS5a, and NS5b, and have a variable function in HCV RNA replication and particle assembly [19].

The core protein, which forms the nucleocapsid, and the envelope glycoproteins (E1 and E2) are the structural components of the virion. The HCV core protein constitutes the viral capsid; it contains a D1 domain responsible for RNA binding and a D2 domain more involved in the association with the endoplasmic reticulum (ER) and with the binding to lipid droplets (LD) membranes [20]. Viral envelope proteins are the principal players mediating virus entry into the host cells [19]. E2 is the receptor-binding protein, interacting with scavenger receptor type B class 1 protein (SR-B1) and tetraspanin CD81, which works as a co-receptor in the adhesion of the virus [21,22]. Meanwhile, E1 protein that is much smaller than E2, is believed to assist E2 to maintain its receptor-binding properties. The E1E2 complex is necessary to interact with the tight junctions protein claudin-1 (CLDN1) essential for HCV entry [21,23]. Recently, E1 has also been believed to serve as the fusion protein between viral lipid envelope and endosomal membrane that will release the HCV RNA genome to the cytoplasm [24].

On the other hand, NS proteins had a complex role in viral replication and particle assembly. Although the functional role is not fully understood, NS2 works as a protease necessary for RNA replication and as a cofactor involved in the viral particle assembly [25]. NS3, together with its cofactor NS4A, mediate the proteolytic release of mature NS4A, NS4B, NS5A, and NS5B, proteins [26]. This processing step is fundamental for viral replication, since in vivo studies revealed that HCV clone lacking NS2/NS3 protease activity is unable to infect a chimpanzee model [27]. NS4B is an integral membrane protein that plays a role in the formation of membranous reticula, a network of small vesicles embedded in a membrane matrix, which represent the site of RNA synthesis and constituting the HCV replication complex [28]. NS4B protein is also involved in the modulation of NS5A hyperphosphorylation [29,30]. NS5A is involved in RNA replication and assembly and has been proved to be involved in resistance to the antiviral activity of IFNα. NS5B is the RNA-dependent RNA polymerase having a key function in viral replication [31]. The ion-channel-forming role of NS1 is not clearly understood, but topological studies have confirmed that NS1 mainly localizes to the ER and is believed to form a proton channel that equilibrates intracellular vesicle pH, creating a favorable environment for virus production [32].

## 3. HCV Core Protein Modulates Multiple miRNAs in Host Cells

The exact mechanism by which HCV infection promotes the development of liver disease remains unclear. However, several studies confirmed the importance of direct interactions of viral proteins with the cellular host machinery in the onset of the disease and its progression to more severe liver damages. Among all, miRNAs have been demonstrated to play fundamental roles in many processes of HCV infection and hepatitis C. It is well documented the importance of the liver-specific miR-122 in the HCV lifecycle and pathogenesis by promoting the viral protein translation, stabilizing the genomic RNA, and inducing viral genomic RNA replication [33,34]. In addition, some interferon (IFN)-induced miRNAs, such as miR-196 and miR-448, have been able to inhibit viral replication by directly targeting the HCV genomic RNA [35]. Besides these two examples of how cellular miRNA can interact with HCV by either promoting or inhibiting viral replication, other recent evidence highlighted the capacity of HCV proteins to modulate the host cell-miRNA expression to establish a favorable environment for its replication and chronic infection.

### 3.1. MiRNAs Induced by HCV Core Protein Inhibit the Interferon Response and Promote Viral Replication

MiR-122 is the most abundant miRNA in the liver, representing 70% of the total miRNA in hepatocytes [36]. MiR-122 enhances the viral replication through the direct interaction with the 5′ noncoding region of the HCV genomic RNA [19]. In addition, it promotes the translation of viral proteins by enhancing the association of the viral RNA with ribosomes [37] (Table 1). Despite miR-122 works as a positive regulator of HCV infection, its persistent and high levels in HCV-infected cells can, in the long term, be deleterious for the establishment of the chronic infection. Indeed, Li S. and colleagues [38] demonstrated that the HCV core protein suppressed the miR-122 expression both in a time- and dose-dependent manner, reducing then Huh7.5.1 cell susceptibility to HCV infection. Thus, suggesting a sort of self-regulatory mechanism of HCV, which may promote the evasion from the cellular immune response and guarantee the persistence of chronic infection. When infected with HCV Jc1 virus, Huh7.5.1 cells showed an increase in both HCV RNA and miR-122. However, at day 19 post-infection, miR-122 dramatically decreased to reach the minimum expression levels at 26–32 days post-infection, corresponding to the plateau in the HCV RNA content [38]. Further experiments evidenced the involvement of HCV core protein in this mechanism. Indeed, the transfection of an enhanced green fluorescent protein (pEGFP)-core expressing plasmid into Huh7.5.1 cells suppressed the miR-122 expression in a time- and dose-dependent manner, with the miR-122 levels proportionally reduced in correspondence to increased amounts of HCV core protein [38]. In parallel, when pEGFP-core-transfected Huh7.5.1 cells were infected with HCV, an inhibitory effect was observed in the viral load at 72 h post-infection, with HCV RNA decreasing in correspondence of the increased amounts of pEGFP-core plasmid transfected into cells [38]. This evidence supports the hypothesis that after the initial phases of the infection, in which the level of HCV RNA increased rapidly, the miR-122 levels are reduced by HCV at later stages. This avoids an over-production of viral particles that might kill host cells, hampering the establishment of a chronic infection. This is supported also by other studies that found an inverse correlation between HCV and miR-122 both in cells and in patients [39]. Subjects with higher viral load had lower miR-122 levels in the liver [40], and the miR-122 expression in serum HCV RNA-positive patients was significantly lower than that in serum HCV RNA-negative patients [41].

Kim and colleagues elucidated the mechanism by which HCV core protein modulates mir-122 expression [42] in cells. They showed that the down-regulation of miR-122 in HCV core-transfected Huh7 cells occurred in a terminal nucleotidyltransferase 2 (TENT2)-dependent manner (Table 1). TENT2, a non-canonical cytoplasmic poly (A) polymerase [53] also known as terminal uridylyltransferase 2 (TUTase-2), catalyzes the tailing of various miRNAs, including miR-122 [53,54]. The HCV core protein was proven to inhibit the 3′-end monoadenylation activity of the TENT2 enzyme resulting in an impaired miR-122 3′ adenylation [42], and thus in an incomplete miR maturation.

Another strongly conserved miRNA, miR-21-5p, was proposed to facilitate HCV replication by counteracting the antiviral activity of IFNα. Indeed, miR-21-5p was proven to target *myeloid differentiation primary response gene 88 (MyD88)* and *Interleukin 1 receptor-associated kinase 1 (IRAK1)*, two factors required for activation of Interferon Regulatory Factor 7 (IRF7), a master regulator of IFNα signaling [55] (Table 1). However, the only experimental evidence of HCV core protein-induced miRNA able to inhibit IFNα cascade was derived from the studies of He CL. and colleagues [44]. In their experiments conducted in Huh7 cells, the HCV-1b core protein was able to increase the expression miR-93-5p, an oncogenic miRNA, often upregulated in HCC tissues [56]. *Interferon receptor 1 (IFNAR1)* was identified as a direct target of miR-93-5p. Since IFNAR1 mediates the IFNα-triggered signal transducer and activator of transcription (STAT) signaling pathway by regulating STAT1 phosphorylation [57], its miR-93-5p-mediated inhibition determines a favorable environment for HCV replication in infected cells [39] (Table 1).

### 3.2. HCV Core Protein Alters Hepatic Lipid Metabolism Through miRNA-Dependent Mechanisms

One of the first studies evidencing the influence of core HCV proteins toward the cellular miRNome, was conducted by Gu Y. and colleagues [58]. The stable transfection in Huh7 cell line of two constructs containing the core proteins of two different HCV genotypes, 3a and 1b, induced the dysregulation of several miRNAs. In particular, they identified 5 up-regulated miRNAs (miR-16-2-3p, miR-423-3p, miR-30a-3p, miR-663 and miR-92b-3p) and 11 downregulated miRNAs (miR-224-5p, miR-629-3p, miR-542-3p, miR-132-3p, miR-455-5p, miR-192-3p, miR-34b-5p, miR-95, miR-885-5p, and miR-664a) in core 3a-transfected cells compared to core 1b-transfected cells [58]. Although with limited experimental evidence, they hypothesized the role of those dysregulated miRNAs in cellular lipid metabolism and apoptosis pathways. It is well known the association of the genotype 3b with hepatic steatosis [59,60]; indeed, the transfection with the core-3b proteins was associated with LD induction, FAS activation, and the alteration of miRNA involved in lipid-related metabolic pathways, such as miR-16-2-3p, miR-30a-3p, miR-34b-5p, miR-92b-3p, miR-132-3p, miR-224-5p [61], miR-664a, and miR-885-5p [58].

In particular, the core protein derived from genotype 3 HCV up-regulates miR-21-5p in infected cell lines (Table 1) [43]. This miRNA has been already associated with HCV infection in patients [39]. Indeed, in a meta-analysis considering a total of 75 HCV patients and 41 controls, the miR-21-5p increase was associated with HCV-positive livers (standardized mean difference 0.65 [95% CI 0.19–1.10, heterogeneity (I2 = 39%)] [43]. In vitro models of Huh-7 cells expressing HCV-3a core, showed a *phosphatase and tensin Homolog (PTEN)* down-regulation mediated by miR-21-5p binding to the 3′UTR [43]. As a consequent effect, cells showed an increase in LD size and accumulation of triglycerides and cholesterol esters, thus suggesting that the PTEN post-transcriptional repression may promote steatosis in the described model [43]. Indeed, PTEN is a master regulator of many pathways in hepatic cells, mainly related to metabolism. Its de-regulation is often associated with many liver metabolic disorders and cancer [62]. This effect was verified in a mir21alox/lox mice (control) and a Mir21a knockout mouse infected with hepatotropic AAV8 encoding for the HCV-3a core under the control of the hepatocyte-specific albumin promoter. Only control mice were able to accumulate LD, with an increased size in hepatocytes expressing high levels of HCV-3a core. On the contrary, high expression of HCV-3a core was unable to trigger enlarged LDs in miR21KO mice [43]. Thus, supporting the evidence that large LD formation is a miR-21-5p-dependent mechanism in HCV-3a core expressing mice.

Several studies have reported the capacity of the HCV core protein to promote lipid accumulation in cell cultures and transgenic mouse models, suggesting that the HCV core protein is sufficient to induce a lipid accumulation in hepatocytes; however, further studies revealed a more articulated mechanism involving miRNAs.

**Figure 2:**
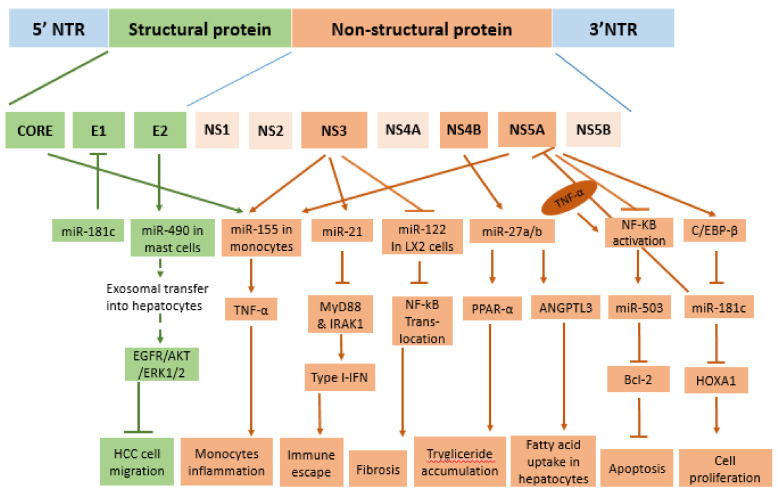
MiRNAs regulated by envelope and non-structural proteins in host cells. E2 proteins induce the release of exosomal miR-490 from mast cells and its internalization by hepatocytes reduced cell migration. NS3 proteins, by modulating miR-155, miR-21, and miR-122, determine inflammation, immune escape, and fibrosis. NS4B, through the modulation of miR-27a and b, affects the lipid metabolism. NS5A inhibits the expression of two oncosuppressor miRNAs, miR-503 and miR-181c.

MiR-27 represents another liver-abundant miRNA, playing a role in lipid metabolism [45] and often deregulated in liver metabolic disorders [45,63]. The two miR-27 isoforms, miR-27a and 27b, have been involved in HCV pathogenesis. In particular, both core and NS4B overexpression in Huh7.5 cells resulted in a Phosphatidylinositol-4,5-bisphosphate 3-kinase (PI3K) pathway-dependent activation of miR-27a/b [45] (Figure 2 and Table 1). As a consequence, in transfected cells, miR-27a/b determined the repression of both Peroxisome Proliferator Activated Receptor Alpha (PPAR-α), which led to triglycerides accumulation and Angiopoietin Like 3 (ANGPTL3), that caused an increase in lipoprotein lipase (LPL) activity and fatty acid uptake into hepatocytes, thus highlighting a possible mechanism contributing to a miR-27a/b mediated HCV-induced steatosis in patients [45]. This mechanism has been further demonstrated in an animal model of acute HCV infection. The SCID-beige/Alb-uPa mice infected with genotype 1a and 2b clinical isolates showed a 2.0-fold and 2.9-fold increase in miR-27a and miR-27b expression at 7 weeks after infection, correlating with the increased cellular lipid content [45]. All these data confirmed the role of both core and NS4B proteins in HVC-induced steatosis, indeed they have already been reported to promote lipogenesis [63] by down-regulating PPAR-α [64] and by increasing sterol response element-binding protein (SREBP) activity through the PI3K pathway [65]. Interestingly, in the same study, the authors observed that miR-27b appear to play an antiviral role against HCV genotype 1b replication. Indeed the PPAR-α down-regulation was able to inhibit genotype 1b HCV replication by inducing hepatic lipid accumulation and blocking the biosynthesis of new lipids required for protein lipidation, and thus efficient HCV replication [66]. In parallel, with the down-regulation of ANGPTL3, miR-27 determined an increased LPL activity that was also responsible of the inhibition of HCV cell-entry [67]. As for miR-122, miR-27 not only appears to have a relevant role for HCV pathogenesis, but also it participates in a HCV self-inhibitory mechanism that ensures adequate levels of viral particles to establish a persistent infection.

The SREBP family of proteins, such as SREBP1a, SREBP1c, and SREBP2, are major regulators of lipid metabolism [68,69] with SREBP2 specifically regulating cholesterol homeostasis [70,71].

HepG2 cells transfected with HCV core 1b protein showed an increase of lipid-associated genes including SREBP1c, SREBP2, HMG-CoA reductase (HMGCR), 3-Hydroxy-3-Methylglutaryl-CoA Synthase (HMGCS), and Sirtuin 1 (Sirt1). Among all, SREBP2 showed a 30% increase compared to controls [46]. In the same cells, miR-185-5p, predicted to target *SRBP2*, was down-regulated. Further gene reporter and miRNA mimic assays proved the capacity of miR-185-5p to bind the SREBP2 3′ untranslated region (3′-UTR) to reduce both SREBP2 mRNA and protein levels. These observations provided evidence of the participation of HCV core protein in the control of cholesterol homeostasis through miR-185-5p inhibition [46] (Table 1). However, the exact mechanism by which this miRNA is down-regulated should be still elucidated.

Further evidence links HCV proteins to miRNAs involved in the regulation of lipid metabolism in liver cells. In particular, miR-758, which is highly expressed in the liver, is involved in lipid and cholesterol metabolism [72], also participating in the cholesterol efflux through posttranscriptional repression of ATP-binding cassette transporter A1 (ABCA1) [73]. High expression of miR-758 was found in patients with HCV infection. Furthermore, the transfection of the HCV core protein in QSG-7701 liver cells induced a 3-fold up-regulation of miR-758 expression compared to controls, within 48 h after transfection [47]. In parallel, miR-758 was shown to target and down-regulate *Toll-like receptor 3 (TLR3)* and *TLR7* in hepatic cells with a subsequent decrease in IFNα and IFNβ [47] (Table 1). Indeed, it has been already hypothesized that HCV may downregulate the two pathogen-recognition receptors TRL3 and TRL7 in chronically infected patients [9,11], and the miR-758, induced by HCV core protein, may play a principal role in this immune evasion mechanisms that ensure the viral persistence.

### 3.3. From Liver Fibrosis to Liver Tumor: The Role of HCV Core Protein in the Dysregulation of Intracellular miRNAs

Approximately 75–85% of HCV-infected patients progress to chronic infection with molecular mechanisms remaining largely unknown [74]. Transforming growth factor-β1 (TGF-β1), one of the main profibrogenic cytokines, regulates the production and accumulation of extracellular matrix molecules (ECMs), stimulating the differentiation of hepatic stellate cells into myofibroblasts [75]. The induction of TGF-β1 is a well-known aspect of HCV infection [75,76,77]. Recently, the HCV core protein was identified as a main player in the TGF-β1 upregulation [48] (Table 1). Indeed, HCV core was able to up-regulate miR-192 in Huh-7 cells [48], which in turn directly repressed the *Zinc Finger E-Box Binding Homeobox 1 (ZEB1*) [78], which is known to decrease the expression of several genes, including *TGF-β1*, with the binding to E-box enhancer element on the promoter [79,80,81]. The absence of ZEB1 reduces the binding of transcription factors such AP-1, Sp1, and Nuclear Factor Kappa B Subunit 1 (NF-kB), which are supposed to activate *TGF-β1* [80,82].

Although chronic inflammation and hepatic cell damages are considered the most common causes of HCV-related carcinogenesis, HCC has been observed in some non-cirrhotic patients with chronic hepatitis C, suggesting a direct involvement of some HCV components to liver carcinogenesis. Indeed, the HCV core protein is considered an oncogenic protein in HCV-related hepatocellular carcinoma [83], being involved in the activation of the Wingless-Type MMTV Integration Site Family (WNT)/b-catenin pathway [84] with effect in proliferation, DNA synthesis, and cell-cycle progression. More recently, the tumor suppressor miR-152 [85,86] was shown to participate in these regulatory mechanisms. In HCV core over-expressing HepG2 cells, the level of miR-152 was significantly lower compared to controls and associated with a significant increase in *Wnt1* mRNA and protein expression [49] (Table 1). The miR-152 down-regulation and the consequent Wnt1 increase had a remarkable effect on the promotion of cell growth, with an increased cell cycle progression from G1 to S phase at 48 h, and in colony formation [49].

These effects were neutralized by the transfection of miR-152 mimic into HCV core over-expressing cells, which bind the 3′-UTR of *Wnt1* RNA, causing a down-regulation of the Wnt/b-catenin pathway [49]. This evidence provides a functional link between the HCV core protein and the acquisition of a malignant phenotype involving the tumor suppressor miRNA-152 and the oncogene *Wnt1*.

Besides, miR-196a also promotes cell proliferation in HCV-infected cells. Indeed, in HepG2 and Huh-7 cells infected with Ad-HCV core adenovirus, the expression of miR-196a was significantly higher compared with the Ad-enhanced green fluorescent protein (EGFP) adenovirus control [50]. Subsequent experiments showed that miR-196 was able to promote G1-S transition, and thus HCC cell proliferation, by inhibiting Forkhead Box O1 (FOXO1) [50], which is a potent transcriptional activator of genes involved in cell cycle arrest, apoptosis, DNA repair, and hypoxia responsiveness [87].

A recent study described the capacity of HCV core protein to induce epithelial-mesenchymal transition (EMT) to enhance HCC aggressiveness [88]. In their study, Liu D. and colleagues showed that HCV core protein was able to induce EMT in normal hepatocytes and HCC tumor cells through the down-regulation of E-cadherin and the up-regulation of EMT markers, such as vimentin, Snail Family Transcriptional Repressor 1 (SNAL1), and SNAL2 [51]. In their experiments, L02 normal human liver cells and HCC-derived HepG2 cells, were infected with an adenovirus-derived construct containing the HCV core protein for 24 h. The transfection resulted in a significant decrease in miR-30c and miR-203a in both cell lines with the concomitant decrease in E-cadherin expression and increase in vimentin, SNAL1, and SNAL2 proteins (Table 1). This miRNA and EMT markers deregulation resulted in increased cell viability and decreased apoptosis susceptibility [51]. Interestingly, the normal L02 hepatocytes, transfected with HCV core protein, acquired tumorigenicity in 40% of inoculated mice, compared to mice inoculated with Ad-blank infected L02 cells [51]. The L02 tumor formation capacity was reduced to 10% in mice inoculated with both HCV-core and miR-30c-expressing constructs. The percentage further diminished to 5% in mice inoculated with HCV-core and miR-203a constructs, thus underlying the tumor-suppressor role of these two miRNAs [51]. Indeed, miR-203 and miR-30c were already proved to target *SNAL2* [89] and *SNAL1* [90,91], respectively, contributing to EMT in cancer. The association with HCV and miR-30c and miR-203a was also evident in patients. MiR-30c and miR-203a levels were significantly lower in HCV positive patients with HCC than in HCV negative patients with HCC [51].

Indefinite growth and suppression of senescence are two hallmarks of cancer. Telomerase reverse transcriptase (TERT) is the main factor responsible for these aspects in the great majority of cancers, including HCC, where it is present in >95% of HCC cases [92]. Recently, Shiu TY. and colleagues showed that the mature HCV core protein, localized in the nucleus, can suppress the expression of miR-138 in Huh7 and HepG2 [52], however, the exact mechanism by which this occurs remains unknown. In cells, the miR-138 transfection decreased the TERT activity, suppressed cell proliferation, and induced cell senescence [52] (Table 1). Thus, the HCV-core-induced miR-138 inhibition was considered as one of the mechanisms able to reactivate TERT expression in HCV-infected cells, possibly leading to cancer development.

## 4. E2 Structural Protein Stimulates the Release of Exosomal miR-490 to Inhibit Cell Migration

HCV envelope proteins, glycoproteins E1 and E2; form heterodimers on viral surfaces; and play an important role in viral infection through the interaction with host cell receptors. Recent studies have identified several miRNAs as key players in virus–host interactions, regulating viral replication and pathogenesis during HCV infection. Only in recent years has the role of HCV E2 in the modulation of cellular miRNAs become evident. HCV-E2 glycoprotein stimulated the production and the exosomal load of miR-490 in mast cells [93]. In their study, Xiong and colleagues showed that exosomal miR-490, released by mast cells, is efficiently internalized by HepG2, where it reduced the activity of epidermal growth factor receptor (EGFR)/protein kinase B (PKB)/ extracellular-signal-regulated kinase1/2 (ERK1/2) pathways, thus inhibiting the migration of cancer cells (Figure 2). Despite this evidence, the mechanisms by which HCV E2 protein modulates miR-490 in mast cells remains unknown. While the role of miR-490 as a tumor suppressor in human cancers is well established [94,95,96], including HCC [97], where it inhibits, metastasis, proliferation, and autophagy [98].

## 5. Non-Structural Proteins in the Regulation of Cellular miRNA Expression

Besides the effects of structural HCV proteins on the expression of miRNAs, some studies also reported the involvement of non-structural proteins in the modulation of miRNAs. In particular, NS3 and NS5 proteins showed numerous interactions with various cellular components that determine the alteration of intracellular miRNAs involved in immune response [55], fibrosis [99], and tumorigenesis [100].

Innate and adaptive immune responses play a fundamental role in host defense against viral infection. In particular, innate immunity represents the first-line defense against the virus. HCV elimination during acute infection corresponds to rapid induction of innate interferon (IFN)-induced genes [101]. However, viruses can develop strategies to evade the type I (IFN)-dependent host immune response [102]. In 2013, Chen and colleagues provided the first direct evidence of the inhibition of type I IFN by miR-21 induced in HCV-infected hepatocytes [55]. In particular, NS5A and NS3/A4 proteins can stimulate the binding of activator protein 1 (AP-1) on the miR-21 promoter, thus determining its up-regulation in infected cells. Interestingly, NS5A regulates miR-21 mainly through the jun proto-oncogene (C-JUN) subunit of AP-1, while NS3/4A through fos proto-oncogene (C-FOS) [55]. NS5A protein induces a phosphorylation cascade that involves protein kinase C epsilon (PKCE) and mitogen-activated protein kinase 8 (MAPK8) to finally activate C-JUN, while NS3/4A activates a phosphorylation cascade that involves protein kinase C alpha (PKCA) and mitogen-activated protein kinase 1 (MAPK1). MiR-21 has been shown to target myeloid differentiation factor 88 (MyD88) and interleukin-1 receptor-associated kinase 1 (IRAK1), which are involved in the HCV-induced type I IFN production [55]. Thus, the inhibition of MyD88 or IRAK1 significantly decreased the mRNA and protein levels of IFN-alpha, providing an escape mechanism from the antiviral immune response of the host cells (Figure 2).

Tumor necrosis factor-α (TNF-α) is a cytokine mediating the immune response to infections, especially against intracellular pathogens [103]. Studies have evidenced the activation of the TNF-α cascade in the chronic HCV infection, with correspondent high levels of serum TNF-α [104]. Recent studies showed a miR-dependent TNF-α induction in HCV infection [105]. In particular, an increase of miR-155 level upon HCV infection was found in circulating monocytes of treatment-naïve patients, which resulted in an acceleration of TNF-α production [106]. Experiments showed that core, NS3, and NS5 proteins were able to induce miR-155 in human monocytes [106]. Indeed, miR-155 functions as a positive TNF-α regulator (Figure 2) [105] in a fine-regulated self-sustaining miR-155/TNFα regulatory loop [107], which ensures high and stable levels of TNF-α during the initial phase of infection. Interestingly, the high miR-155 expression in the serum of HCV patients correlated with the high levels of serum of miR-122, thus suggesting a possible role of this miR as a marker of inflammation-induced hepatocyte damage [106]. However, the effects of the increased circulating TNF-α levels were mitigated in liver cells by the activity of NS5A protein. Indeed, in HepG2 cells, NS5A protein inhibited TNF-α-induced NF-κB activation in a dose-dependent manner, although with an unknown mechanism [108].

NF-κB was proved to bind the miR-503 promoter to promote its transcription [109]. Thus, the NF-κB inhibition determined by the NS5A overexpression in HepG2 cells caused a miR-503 reduction and a consequent up-regulation of its target, B-cell lymphoma 2 (BCL-2), resulting in a reduction of apoptosis (Figure 2) [108].

Liver fibrosis is the excessive accumulation of extracellular matrix (ECM) proteins and is considered as one of the clinical complications of chronic hepatitis C infection without treatment. Some experimental evidence highlighted the involvement of NS3 protease in the initiation and progression of fibrosis [110,111], also with the implication of miRNAs.

In an exploratory study, Khanizadeh and collaborators observed the alteration of some miRNAs in LX-2 cells transfected with NS3 protein. In particular, miR-335 and miR-150 were dramatically downregulated while miR-27a expression was upregulated [99]. Interestingly, those miRNAs have a validated role in the progression of liver disease. Indeed, miR-335 [112] and miR-150 [113] have an anti-fibrotic activity in cells while miR-27 acts as a pro-fibrotic miRNA [114]. When overexpressed in LX-2 cells, miR-150 inhibited the activation of hepatic stellate cells (HSCs) by targeting the *myeloblastosis transcription factor (C-MYB)*, which is responsible for the production of smooth muscle-actin (SMA) and collagen type I [113]. In addition, in HSCs, miR-335 inhibits tenascin-C (TNC), an ECM glycoprotein involved in cell migration [112]. By contrast, the pro-fibrotic miR-27a and 27b promoted cell proliferation during HSCs activation by targeting retinoid X receptor a (RXRa), a transcription factor for multiple genes involved in cell proliferation and differentiation [114].

Further investigations by Khanizadeh and collaborators noticed that NS3 protein, retaining the wild-type protease activity, was able to induce pro-fibrotic genes in LX-2 cells, such as α-SMA, Collagen type 1A (COL1A), and Tissue Inhibitor of Metalloproteinases 1 (TIMP-1), as well as the release of transforming growth factor-beta (TGF-β1) [115]. On the contrary, no or limited effect was observed in cells transfected with NS3 protein defective of the protease activity [115]. Moreover, both wild-type and mutated NS3 downregulated intracellular levels of miR-122, possibly contributing to hepatic fibrosis. Indeed, miR-122 possesses anti-fibrotic properties by blocking the NF-κB translocation in LX-2 cells (Figure 2). Also, it negatively regulates the production of the inflammatory cytokines IL-6, (Monocyte Chemotactic Protein 1) MCP-1, and IL-1β by targeting PKR activating protein (PACT), an important molecule for the production of interferon and cytokines [116].

The progression of liver fibrosis through more severe stages corresponds to an increased risk for the development of liver cancer.

In 2015, Zhang and colleagues studied the miRNome of HepG2 cells transfected either with a plasmid expressing the full-length NS3 protein or the NS3/4A protein. A total of 35 miRNAs were dysregulated, NS3 expressing HepG2 while 75 miRNAs were altered in HepG2 NS3/4A cells [100]. With the exceptions of miR-143, miR-181c, miR-181d, and miR153, the NS3 and the NS3/4A-expressing HepG2 had a differential miRNA profile with miR-130a and miR-153 being the most significantly altered miRNAs in HepG2-NS3 and HepG2-NS3/4A cells, respectively. The differences in the miRNA profiles of HepG2-NS3 and HepG2-NS3/4A may explain the differential cellular behavior, HepG2-NS3 having a more marked colony formation capacity and tumorigenicity in vivo than HepG2-NS3/4A cells [100]. Indeed, the up-regulation of miR-122, a well-known oncosuppressor, was only observed in NS3/4A-expressing cells [100]. MiR-122 represses HCC by targeting multiple genes involved in carcinogenesis [117], such as *cyclin G1* [118] and *ADAM Metallopeptidase Domain 17 (ADAM17)* [119], involved in proliferation and EMT, respectively.

Mukherjee and colleagues (2014) described the potent antiviral activity of miR-181c against HCV since it can directly target the HCV envelope glycoprotein E1 and the nonstructural protein NS5A coding sequences. Indeed, exogenous miR-181c determined a downregulation of viral replication when introduced into Huh7.5 cells [120]. As an escape mechanism, HCV evolved the capacity to indirectly inhibit miR-181c expression in infected cells. Particularly, NS5 protein was able to suppress the activity of the transcription factor CCAAT/enhancer-binding protein β (C/EBP-β) C/EBP-β, which binds to the miR-181c promoter to stimulate its expression (Figure 2) [120]. One of the cellular targets of mir-181c is *homeobox A1 (HOXA1)*, which encodes for a transcription factor involved in cell proliferation and tumoral transformation, thus by inhibiting miR-181c, HCV may also be responsible for the oncogenic role of the virus in hepatic cells. Interestingly, these results remain consistent with the observations of Zhang and colleagues that reported downregulation of miR-181c in HepG2 cells transfected either with NS3 protein or NS3/4A protein, thus evidencing the importance of a miR-181c inhibition in infected cells. However, it is not clear what the advantage is of this downregulation for the virus [100].

## 6. The Role of HCV-Induced miRNAs in Liver Carcinogenesis

The exact mechanism by which HCV induces carcinogenesis remains largely unknown. The persistence of inflammation is one of the main factors contributing to hepatocarcinogenesis, and the NF-κB pathway represents a key component linking liver injury, fibrosis, and HCC. However, what is clear is that viral proteins disrupt host cell signals in transduction pathways that affect cell proliferation, cell survival, and lately transformation, possibly by de-regulating host cell miRNAs.

### 6.1. HCV Proteins Determine the Up-Regulation of miRNA Involved in Liver Cancer Development

MiR-21 was one of the first identified oncomiRs usually over-expressed in multiple solid cancers, including HCC [121]. This miRNA has been linked to proliferation, migration, and invasion of hepatoma cells through direct interaction with PTEN, a tumor suppressor [122], and kruppel like factor 5 (KLF5) [123]. Upon HCV infection, miR-21 upregulation was found in hepatocyte-like cells and liver biopsies of chronic patients [39]. Besides, it is involved in the pathogenesis of liver fibrosis in HCV-infected patients through the modulation of a small mother against decapentaplegic homolog 7 (SMAD7) and TGF-β signaling pathways [39]. Interestingly, studies have shown its potential as a circulating biomarker in different phases of the disease. Plasma miR-21 had a strong correlation with fibrosis levels in patients infected with HVC, and the increased levels, together with vascular endothelial growth factor (VEGF) and alpha fetoprotein, can serve as an early biomarker for HCV-derived HCC [124]. Consistently, increased levels of miR-21 were observed in sera and tissues of patients with cirrhosis and HCC with poor prognosis [125].

Using a microarray to profile the changes in the miRNA expression throughout different stages of liver tumorigenesis, Pineau and colleagues identified a set of 12 miRNAs. Among them, miR-93 resulted in being significantly dysregulated from normal to cirrhosis and HCC [126]. Subsequently, several studies described the oncogenic role of this miRNA in HCC. As for miR-21, the principal role of miR-93 is the promotion of cell proliferation by targeting *transforming growth factor β type II (TGFBR2)* and *integrin beta 8 (ITGB8).* Indeed, its inhibition significantly reduced the cell turnover and colony formation of cultured HepG2 and primary human hepatocytes [127]. Also, Ohta’s study identified *PTEN* and *Cyclin-Dependent Kinase Inhibitor 1A (CDKN1A)* as two targets of miR-93 involved in cell proliferation and survival. Their inhibition led to the activation of the c-MET/PI3K/AKT cascade that reduced cell apoptosis in different malignant cells [128]. In parallel, miR-93 can inhibit Mitogen-Activated Protein Kinase Kinase Kinase 2 (MAP3K2), which activates the downstream c-Jun N-terminal kinases (JNK) pathway to promote cell cycle progression [129]. Due to its role in cancerogenesis, miR-93 has been suggested as an indicator of poor prognosis in HCC [128].

Other miRNAs frequently upregulated in HCC are miR-27a and miR-27b [130]. By targeting *Sprouty RTK Signaling Antagonist 2 (SPRY2)*, which was previously described to suppress the AKT/MAPK pathway-induced hepatocarcinogenesis in mouse liver [131], miR-27b promoted cell migration and invasion [132]. Similarly, the suppression of PPAR-γ expression by miR-27adetermined a cell cycle progression and an inhibition of apoptosis in HepG2 cells [130].

Suppressor of cytokine signaling 2 (SOCS2), a member of the SOCS family, is down-regulated in aggressive tumors and correlated with poor prognosis of HCC patients [133]. In a study on SMMC-7721 and HepG2 cells, miR-196 was found to attenuate the transcriptional activity of SOCS2 factor, thereby increasing the phosphorylation of Janus kinase 2 (JAK2) and Signal Transducer and Activator of Transcription 5A (STAT5) proteins, which play a vital role in lipid metabolism and cancer development in the liver [134,135]. Indeed, HCC patients expressing high miR-196 levels exhibited significantly greater macrovascular invasion than patients having a lower expression [136].

### 6.2. The Down-Regulation of Host Cell miRNAs Contribute to Liver Carcinogenesis

**Figure 3:**
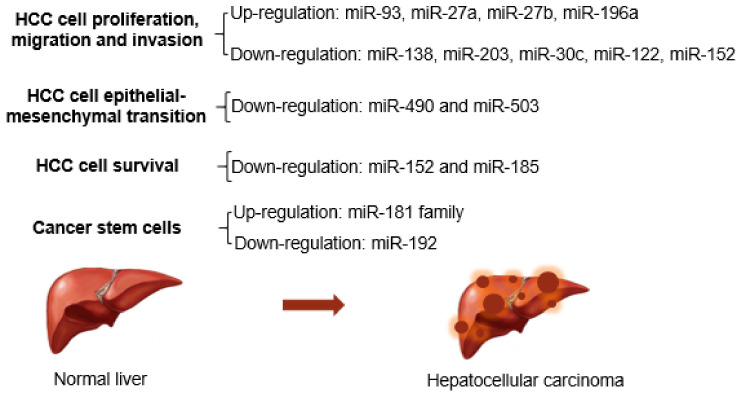
List of miRNA participating in cancerogenesis. HCV proteins regulated several miRNAs that are involved in multiple oncogenic processes.

The liver-specific miR-122 is a highly expressed miRNA that accounts for 70% of total liver miRNAs [137]. It plays an important role as an anti-tumor miRNA and may represent a potential biomarker for the detection of liver damage and HCC progression [138]. It is well established the importance of miR-122 during HCV infection [33,139], as well as in the inhibition of HCC [117]. It participates in multiple cellular pathways involved in hepatocarcinogenesis and EMT (Figure 3). MiR-122 promotes P53 expression by targeting *Cyclin G1*, a negative regulator of P53 [140]. In addition, miR-122 suppresses insulin growth factor 1 receptor (IGF-1R), reducing the IGF-1R/AKT signaling, which sustains glycogen synthase kinase-3 beta (GSK-3β) activity, thus resulting in a repression of cyclin D1 expression and cell proliferation [141]. Another direct target of miR-122 is *WNT1*, thus miR-122 works as a suppressor of the WNT/β-catenin signaling pathway [142].

The low expression of miR-185 has been associated with a high rate of tumor recurrence and poor prognosis of patients at the early HCC stage [143]. In cells, miR-185 suppresses cancer formation by inducing cell cycle arrest and apoptosis by targeting *Ras Homolog-MTORC1 Binding (RHEB), RPTOR Independent Companion of MTOR Complex 2 (RICTOR)*, and *AKT Serine/Threonine Kinase 1 (AKT1)* [144].

Indeed, RHEB is an upstream activator of mammalian target of rapamycin complex 1 (mTORC1), which can, in turn, promote the production of several angiogenesis-related proteins, such as hypoxia-inducible factor α (HIF-α) and VEGF. While RICTOR is an essential component of the mTORC2 complex, which has a role in the phosphorylation of AKT/protein kinase B (PKB) whose activation leads to cell proliferation and survival [145]. Thus, by inhibiting the two complexes of the mTOR pathway, miR-185 demonstrated its pivotal function as a tumor suppressor in HCV-induced HCC (Figure 3).

Additionally, miR-185 can inhibit HCC development by suppressing DNMT-1, PTEN, and AKT functions [146].

Although much of its function in HCC remains to be explored, the high expression of miR-758 was observed in HCC; interestingly, a subset of HCC patients under 66 years old had higher miR-758 levels, compared to elder patients [147].

HCV core protein has been shown to increase the cellular levels of miR-192 [48], however, in HCC, it seems to work as a tumor-suppressor miRNA through a direct impact on the function of the SLC39A6 transporter, and SNAIL pathway [148]. Moreover, silencing of miR-192 by hypermethylation is one of the key drivers of liver carcinogenesis through the acquisition of stemness features of hepatocytes [149]. Consistently, miR-192 has been used as a prognostic marker in HCC since its down-regulation has strongly anticipated cancer metastasis [148].

HCV core protein inhibited miR-152 expression and subsequently enhanced the expression of WNT1 in HepG2 cells [49]. Furthermore, in HCV-induced liver cirrhosis, miR-152 was proposed as a biomarker of poor outcomes in the patients who expressed a relatively low level of this miRNA [150]. Indeed miR-152 is considered a tumor-suppressor miRNA. The miR-152 transfection in various HCC cell lines determined the inactivation of ERK and AKT pathways and significantly suppressed cell proliferation and motility while inducing apoptosis [151]. *Rhotekin (RTKN)*, a tumor-promoting protein, was also confirmed as a direct target of miR-152 to reduce tumor development [152].

Hypermethylation-mediated miR-203 silencing was detected in primary HCC cases compared with healthy controls [153]. Besides the targeting of ATP Binding Cassette Subfamily E Member 1 (ABCE1) factor, involved in tumor growth [153], miR-203 also modulated the oncogene ADAM Metallopeptidase Domain 9 (ADAM9) and long non-coding RNA HULC to inhibit the invasion and migration of cancer cells [154]. Moreover, it suppressed the anti-apoptotic protein Survivin to inhibit HepG2 proliferation [155], and the transcription factor Homeobox D3 (HOXD3), hampering the metastatic process and angiogenesis of various cancer cells [156] (Figure 3). Interestingly, HCV core protein has been reported to down-regulate both the expressions of miR-203 and miR-30c to activate EMT pathway-associated in the development of liver cancer [51].

MiR-30c holds the potential as a non-invasive marker for the early detection of liver cancer in HCV-infected patients [157]. As a tumor-suppressor miRNA, miR-30c suppresses cell growth by inhibiting TGF-beta-induced Serpine 1 [158] and/or B-Cell CLL/Lymphoma 9 Protein (BCL9) [159] (Figure 3). Besides the HCV-core-induced downregulation, miR-30 can be sponged by the lncRNA-CCAT1 to prevent the *Cyclin E1* targeting and thus promote cancer cell proliferation and invasion [157]. However, it remains to be addressed if the different mechanisms involved in miR-30c downregulation occur simultaneously in cells.

By investigating the miRNA dysregulation in tumors derived from HCC patients, Wang’s group identified miR-138 as a frequently down-regulated miRNA in tumor samples [160]. MiR-138 has anti-tumoral activities by targeting *Forkhead box C1 (FOXC1)* [161], *Cyclin D3* [160], and *SRY-Box Transcription Factor 9 (SOX9)*. The low miR-138 levels in tissues were considered as an independent prognostic factor related to patients’ survival [162].

MiR-490 was reported to diminish the metastasis of different HCC cell lines by reducing the transcriptional levels of E2F Transcription Factor 2 (E2F2) and Epithelial Cell Transforming Sequence 2 (ECT2) factors. An abnormal level of miR-490 was also associated with the poor prognosis of HCC patients [163]. An inhibition of BUB1 Mitotic Checkpoint Serine/Threonine Kinase (BUB1) by miR-490 was observed in various in vitro models resulting in a modulation of TGFβ/SMAD signaling pathways to reduce HCC cell proliferation, migration, as well as invasion [164]. The role of miR-490 as a tumor suppressor was also proved by its role in the inhibition of the aurora kinase A (AURKA), which is a member of a family of mitotic serine/threonine kinases often associated with high occurrence of cancer [165].

By suppressing miR-503, the NS5A protein may contribute to hepatocarcinogenesis [108]. Indeed, the anti-tumor miR-503 suppressed the progression of HCC by targeting the *protein arginine methyltransferase 1 (PRMT1)* and the *WEE1 G2 Checkpoint Kinase (WEE1)* to block EMT in HepG2 cells [166,167]. Interestingly, the induction of miR-503 promoted the susceptibility of HCC cells to a 5-fluorouracil anticancer drug [168].

In searching for miRNAs correlating with liver cancer stem cells, Wang’s group identified the miR-181 family playing a pivotal role in maintaining the stemness feature of a subset of HCC cells. In particular, the miR-181 family directly targeted several factors namely *caudal type homeobox transcription factor 2 (CDX2)*, *GATA binding protein 6 (GATA6)*, and *Nemo-like kinase (NLK)*. In contrast, miR-181c was reported to inhibit HCC progression through regulating the function of Non-SMC Condensin I Complex Subunit G (NCAPG) in HCC cell lines SMMC-7721 and MHCC-97H [169]. Thus, by suppressing miR-181c, NS5 protein may be responsible for the oncogenic role of the virus in hepatic cells [120].

## 7. Conclusions

As fundamental regulators of gene expression, miRNAs became part of the HCV-host cell interaction network that leads to cell reprogramming to foster viral replication and immune system escape. Consequent to these perturbations, some oncogenic pathways were triggered. Despite DAA holding promise in the complete eradication of HCV worldwide, the issue of HCC occurrence and recurrence after HCV eradication remains, and miRNAs de-regulated by HCV may be the key players to look at, even in terms of possible biomarkers for an early HCC detection.

## Figures and Tables

**Table 1:** List of cellular miRNA regulated by HCV core protein.

miRNA	HCV Protein	miR-Expression	Cell Model	Animal Model	Effect
miR-122 [38,42]	Core	Down	Huh7.5.1, Huh7		Positive regulator of infection at initial phases. The Inhibition of TENT-2, by core protein, affects the miR-122 maturation and function. HCV self-inhibitory effect.
miR-21 [43]	Core Genotype 3a	Up	Huh-7	mir21a^lox/lox^ (control) and Mir21a KO mice	Downregulates PTEN to promote lipid accumulation and steatosis. Promote viral replication.
miR-93 [44]	Core Genotype 1b	Up	Huh7		IFNAR1 down-regulation to abolish IFN α pathway.
miR-27a and miR-27b [45]	Core Genotype 1b	Up	Huh7.5	SCID-beige/Alb-uPa mice infected with genotype 1a and 2b	Repression of PPAR-α, which led to triglycerides accumulation, and ANGPTL3, which caused an increase in lipoproteinlipase activity and fatty acid uptake. HCV self-inhibitory mechanism
miR-185 [46]	Core Genotype 1b	Down	HepG2		Targets SREBP2, control of cholesterol homeostasis.
miR-758 [47]	Core	Up	QSG-7701		Regulation of the cholesterol metabolism, also controlling the cholesterol efflux through ABCA1 repression. TLR3 and TLR7 downregulation with a subsequent decrease in IFNα and IFNβ.
miR-192 [48]	Core Genotype 1b	Up	Huh-7, Huh-7.5		Downregulates ZEB1 responsible for the TGF-β1 inhibition.
miR-152 [49]	Core	Down	HepG2		WNT1 increase with a consequent promotion of cell growth and colony formation.
miR-196a [50]	Core Genotype 1b	Up	HepG2, Huh-7		FOXO1 down-regulation with a consequent proliferation.
miR-203 [51]	Core Genotype 1b	Down	L02 normal human liver, HepG2	Balb/C nude mice injected with L02 or HepG2	Induced EMT, increase in cell viability, and a decreased apoptosis susceptibility, possibly by up-regulating SNAL2, the miR-203 target.
miR-30c [51]	Core Genotype 1b	Down	L02 normal human liver, HepG2	Balb/C nude mice injected with L02 or HepG2	Induced EMT, increase in cell viability, and a decreased apoptosis susceptibility, possibly by up-regulating SNAL1, the miR-30c target
miR-138 [52]	Core Genotype 1b	Down	HepG2, Huh-7		TERT increased expression. Indefinite growth and suppression of the senescence process.

## Appendix A

A literature search was performed by four independent researchers in PubMed database, by using the keywords “HCV”, “microRNA”, “HCV proteins”, “miRNA” “HCC”. Only studies reporting clear evidence of HCV proteins modulating host miRNA expression were included in the literature review.
